# New combinatorial strategies to improve the PARP inhibitors efficacy in the urothelial bladder Cancer treatment

**DOI:** 10.1186/s13046-019-1089-z

**Published:** 2019-02-22

**Authors:** Daniela Criscuolo, Francesco Morra, Riccardo Giannella, Roberta Visconti, Aniello Cerrato, Angela Celetti

**Affiliations:** 1 Institute for the Experimental Endocrinology and Oncology, Research National Council, CNR, Naples, Italy; 2 Urology Surgery Unit, A.Cardarelli Hospital, Naples, Italy

**Keywords:** DNA damage response, Synthetic lethality, BRCAness, CCDC6, Biomarkers, Immunotherapy, Epigenetic agents, PARP trapping, RRx-001, Viral mimicry

## Abstract

**Background:**

Novel therapeutic strategies are urgently needed for the treatment of metastatic Urothelial Bladder Cancer. DNA damaging repair (DDR) targeting has been introduced in cinical trials for bladder cancer patients that carry alterations in homologous DNA repair genes, letting to envisage susceptibility to the Poly (adenosine diphosphate [ADP]) ribose polymerase (PARP) inhibitors.

**Main body:**

PARP inhibition, by amplifying the DNA damage, augments the mutational burden and promotes the immune priming of the tumor by increasing the neoantigen exposure and determining upregulation of programmed death ligand 1 (PD-L1) expression. Thus, the combination of PARP-inhibition and the PD/PD-L1 targeting may represent a compelling strategy to treat bladder cancer and has been introduced in recent clinical trials. The targeting of DDR has been also used in combination with epigenetic drugs able to modulate the expression of genes involved in DDR, and also able to act as immunomodulator agents suggesting their use in combination with immune-checkpoint inhibitors.

**Conclusion:**

In conclusion, it may be envisaged the combination of three classes of drugs to treat bladder cancer, by targeting the DDR process in a tumor context of DDR defect, together with epigenetic agents and immune-checkpoint inhibitors, whose association may amplify the effects and reduce the doses and the toxicity of each single drug.

## Introduction

Bladder cancer (BC) is the ninth most common malignacy disease worldwide. Urothelial bladder cancer (UBC) represents the prevalent histological type of BC at least in the United States and in Europe. Among newly diagnosed patients, approximately 70% present with a non-muscle invasive bladder cancer (NMIBC), while 30% of UBC patients present with a muscle-invasive (MIBC) or a metastatic disease (mUBC) [1]. The current standard of care for patients with locally advanced and metastatic urothelial bladder cancer is cisplatin-based combined chemotherapy [2]. However, almost half of patients show recurrence or progression of the disease and about one-third of patients are not eligible for first-line cisplatin-based therapy due to comorbidities [3, 4]. Until recently, the management of mUBC has not changed significantly. Notably, in 2016, the approval of immune checkpoint inhibitors (ICIs) for the treatment of patients with advanced bladder cancer who are refractory or ineligible to platinum-based chemotherapy, has improved the course of this deadly disease [5]. Immune-checkpoint inhibitors by targeting the pathways that cancer cells use to evade the host immune system promote a significant anti-tumor activity. However, only 20–30% of patients with mUBC achieve a partial or complete response to immune-checkpoint inhibitors. Therefore, the identification of new therapeutic strategies for the treatment of mUBC remains a critical focus. Recently, the synergistic combination of immune checkpoint inhibitors with DNA damage response targeting agents or with epigenetic drugs has been proposed for the treatment of different tumors including mUBC [6, 7].

In this review, we intend to describe the emerging role of defects in DNA damage response and repair (DDR), as cause of genome instability and possible target of therapy in mUBC, by inhibiting enzymes involved in the repair of single strand breaks, such as the Poly (adenosine diphosphate [ADP]) ribose polymerase (PARP). Moreover, we also analyse how the accumulation of damage to the DNA may lead to immune-priming effects in tumor cells promting the response to immune-checkpoint inhibitors. In this way, the targeting of DDR combined with immunotherapy has the potential to expand and heighten the cancer patients responses, as supported by the results reported in recent clinical trials, which combine PARP-inhibitors and immunotherapy. Interestingly, the targeting of DDR has been combined with epigenetic drugs, able to modulate the expression levels of genes involved in DDR process, and acting also as immunomodulatory agents, suggesting a possible use in combination with immune checkpoint inhibitors.

Finally, we discuss the possibility to combine three classes of drugs to treat bladder cancer, by targeting the DDR process in a tumor context of DDR defect, together with epigenetic agents and immune-checkpoint inhibitors, whose association may amplify the effects and reduce the doses and the toxicity of each single drug.

## Rationale for the use of poly (ADP-ribose) polymerase inhibitors in the treatment of urothelial bladder cancer

### DNA damage response as a therapeutic target

**Fig. 1:**
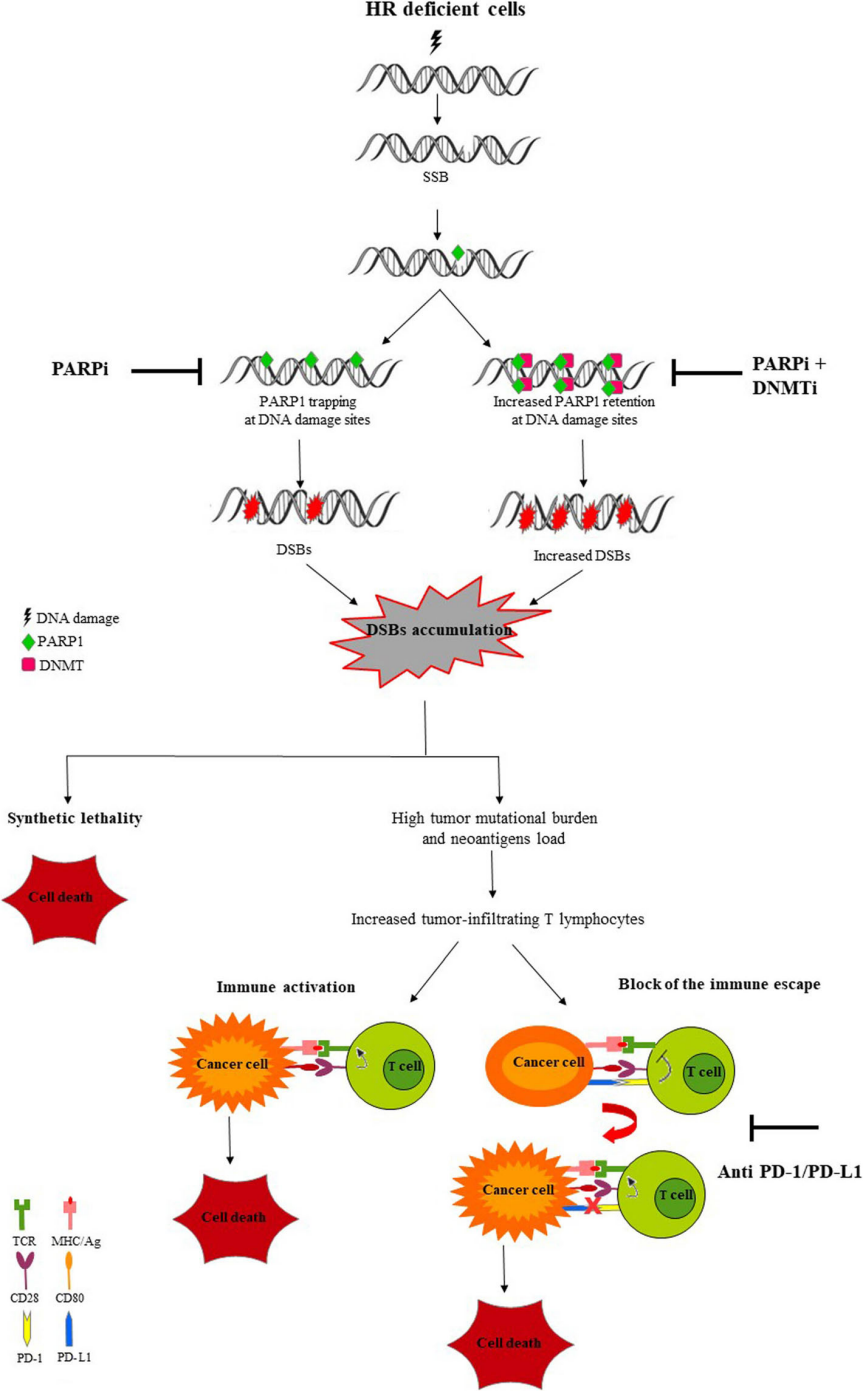
The homologous recombination (HR) repair deficiency represents an opportunity for a synthetic lethality approach through the use of PARP inhibitors (PARPi). The DNA methyltransferase inhibitors (DNMTi) enhance the cytotoxic effects of PARPi by increasing the PARP1 trapping at DNA. At the same time, the accumulation of DNA damage, as a consequence of cell’s inability to repair the DSBs, results in a high tumor mutational burden and tumor surface neoantigens load associated with increased infiltration of T lymphocyte into tumor microenvironment. These events trigger the compensatory upregulation of PD-1/PD-L1 pathway offering the possibility to use the immune checkpoint inhibitors in order to kill the cancer cells that elude the immune system. Thus, targeting the PD-1/PD-L1 pathway with immune checkpoint inhibitors may rappresent an attractive approach for treament of the tumor with defects of HR repair

The human genome is continuously exposed to a wide range of potential sources of damage. In order to face these attacks, the cells have evolved a complex signaling pathway, called DNA damage response (DDR), that senses DNA damage and promotes the maintenance of genome integrity [8]. Defects in one of the components of the DDR network lead to genomic instability, one of the hallmarks of cancer [9]. At the same time, DDR targeting represents an attractive therapeutic strategy especially effective in cells that already carry a DNA repair gene defect [10]. The paradigmatic example of DDR targeting is represented by the PARP inhibitor Olaparib (Lynparza), recently FDA approved as single agent for treatment of breast and ovarian cancers harboring BRCA1 or BRCA2 germline mutations, i.e. carrying defects in DNA repair by homologous recombination (HR) [11]. The antitumor activity of PARP inhibitors in HR-deficient tumors is based on the concept of synthetic lethality, a perturbed status of the cell in which two genes/pathways when affected simultaneusly lead to cell death [12]. In the case of PARP inhibitors the presence of a loss of function mutation in a HR-related gene associated with pharmacological inhibition of a protein involved in a complementary DDR-pathway, such as PARP, leads to genomic instability and cell death (Fig. 1) [13]. In particular, Poly(ADP-ribose) polymerase (PARP)-1 enzyme, a PARP family member, plays an important role in the repair of DNA single strand breaks (SSBs), which can be generated during base excision repair (BER) [14]. The PARP1 enzyme binds to DNA single strand and catalyzes, by using nicotinamide adenine dinucleotide (NAD+) as a substrate, the transfer of poly ADP-ribose (PAR) polymers to proteic residues on acceptor proteins, including PARP1 itself [15]. This process of “PARylation” allows the recruitment of DNA repair proteins at DNA break-sites. Auto-PARylation of PARP1 leads to its dissociation from DNA, which is required for the completion of DNA repair [16]. The PARP inhibitors act by competing with NAD+ for binding to the catalytic domain of PARP, inhibiting PARylation and trapping PARP to the damaged DNA, thus preventing the SSBs repair that degenerate into DNA double strand breaks (DSBs) [17, 18]. When PARP activity is pharmacologically inhibited in HR-deficient cells, the DSBs can be repaired only through non-homologous end joning (NHEJ), an error prone pathway, leading to genome instability and cell death [16]. Our current knowledge suggests that PARP inhibitors may have a wider application. Indeed, several tumors carrying somatic mutations in DDR genes, other than BRCA1 or BRCA2, including ATM, ATR, BARD1, BRIP1, CHK1, CHK2, PALB2, RAD51 and FANC, might exhibit a phenotype known as BRCAness and thus benefit from PARP inhibitors treatment [19]. Recently, PARP-inhibitors drugs have been approved for prostate cancer treatment [20] and introduced in several clinical trials for additional tumors that exhibit DNA-repair defects, including bladder cancer (NCT03375307) [Table 1].

**Table 1:** Ongoing phase I/II studies testing PARP-inhibitors, immune checkpoint inhibitors and epigenetic drugs as monotherapy or in combinatorial regimen in advanced urothelial cancer

ClinicalTrials.gov identifier	Phase	Patients	PARP inhibitor	Checkpoint inhibitor	Epigenetic drug	References
NCT03448718	II	Metastatic Urothelial Cancer Harboring DNA Damage Response Gene Alterations	Olaparib			N/A
NCT03375307	II	Metastatic or Advanced Urothelial Cancer With DNA-Repair Defects	Olaparib			N/A
NCT03397394 (UCLA)	II	Locally Advanced or Metastatic Urothelial Carcinoma	Rucaparib			N/A
NCT02736266	II	Muscle-invasive Urothelial Bladder Carcinoma		Pembrolizumab		N/A
NCT02951767 (IMvigor 210)	II	Locally Advanced or Metastatic Urothelial Bladder Cancer		Atezolizumab		36
NCT02108652 (IMvigor 211)	II	Locally Advanced or Metastatic Urothelial Bladder Cancer		Atezolizumab		79
NCT02546661 (BISCAY)	Ib	Muscle Invasive Bladder Cancer	Olaparib	Durvalumab		N/A
NCT03534492 (NEODURVARIB)	II	Prior to Surgery of Resectable Urothelial Bladder Cancer	Olaparib	Durvalumab		N/A
NCT03459846 (BAYOU)	II	Advanced, Platinum-Ineligible Bladder Cancer	Olaparib	Durvalumab		N/A
NCT02619253	I	Advanced renal or urothelial cell carcinoma		Pembrolizumab	Vorinostat	N/A
NCT03179943	II	Advanced, Platinum-Ineligible Bladder Cancer		Atezolizumab	Guadecitabine	N/A

### DNA damage repair deficiency in urothelial bladder Cancer

Defects in DNA repair genes predict response to neoadjuvant cis-platin-based chemotherapy in muscle-invasive bladder cancer [21]. Somatic mutations in ERCC2, a member of the nucleotide excision repair pathway, conferred vulnerability to cisplatin chemotherapy in this tumor. No relations have been reported between ERCC2 mutations, or other NER members alterations, with PARP inhibitors sensitivity. Nevertheless, a relation between ERCC2 mutations or ERCC2 low levels have been correlated to cisplatinum sensitivity and PARP-inhibitors resistance in ovarian cancer [22]. Interestingly, alterations in the DDR genes ATM, RB1 and FANCC have been reported as biomarkers of platinum sensitivity in bladder cancer and few preclinical data have shown that antitumor activity of PARP inhibitors in combination with cisplatin may determine a significant increase in DNA damage versus use of cisplatin alone in urothelial bladder cancer [23]. The comprehensive genetic characterization of muscle-invasive bladder cancer have recently reported loss of function mutations in several DDR genes, such as CHK1/2, RAD51, BRCA1/2, ATM, ATR, MDC1 and FANCF, identified in 34% of tumors [24, 25], suggesting the possibility of using the PARP inhibitors for advanced UBC treatment. A clinical trial investigating the efficacy of the PARP inhibitor Olaparib, as single drug, for treating patients with mUBC and DNA-repair genes defects, has been recently launched (NCT03375307) and estimated to be completed by December 2022 [Table 1]. In order to enlarge the number of bladder tumors that could benefit from the PARP inhibitors treatment it is extremely urgent to identify novel biomarkers able to detect HR-DNA repair defects in tumor specimens and to predict the response to DDR targeting. A transcriptional signature of DNA repair deficiency has been investigated in germline and sporadic BRCA1/2 positive breast cancers, as well as the development of HR assay is highly pursued [26]. On this matter, few companies have introduced novel tests to screen germline mutated BRCA ovarian cancer, or triple negative or BRCA-mutated breast patients for the HR DNA repair ability, evaluating tumour sequencing and DNA cytogenetics or assigning a score upon assessing the LOH (loss of heterozygosity), the telomeric allelic imbalance and the large-scale state transition, provided by the Myriad HRD test [27]. Currently, with the aim to identify predictive biomarkers and to select PARPi candidates for neoadjuvant application also in combination with cisplatinum, clinical studies are underway to validate the accuracy of these tools to predict HR defects (HRD), chemotherapy resistance and PARPi sensitivity also in patients with genitourinary malignancies, such as prostate cancer and bladder UC [27]. In preclinical studies, we have recently reported that cells defective for CCDC6 perform as BRCA-like cells, with resistance to chemotherapeutic agents and sensitivity to small molecule inhibitors of the repair enzymes PARP1/2 [28]. The tumor suppressor CCDC6 has a prominent role in the DNA damage response and can influence genome stability in primary tumors [29], being as other genes involved in DDR pathways often deregulated or inactivated in tumors [30]. CCDC6 has been reported to negatively modulate the catalitic subunit of the serin-threonin protein phosphatase 4 (PP4c) determining the H2AX activation upon stress [31]. Recently, low levels of CCDC6 have been reported to be associated with an impairment of HR mechanisms, in lung, colon and prostate cancer models affecting cells behavior and cells sensitivity to PARP inhibitors (PARPi) [32, 33]. CCDC6 attenuation in these cancer cells confers resistance to cisplatin and sensitizes the cells to the PARPi olaparib, and the combination is more effective than each agent individually [34, 35]. In bladder urothelial cancer, low levels of CCDC6, accompanied by HR-DNA repair defects might indicate the use of PARP inhibitors treatment (Morra F, Merolla F, Criscuolo D et al., JECCR, accepted for publication), while the chemosensitivity in CCDC6 deficient/proficient bladder tumors still is under investigation. Thus, the paradigm of synthetic lethality, in support of the use of PARP inhibitors drugs may help the management of mUBC, a deadly disease whose outcome could be ameliorated by the identification of predictive biomarkers of HR defects.

### PARP-inhibitors and immune-checkpoint inhibitors combination

Since 2016, five immune checkpoint inhibitors (ICIs) have been introduced for mUBC as second-line treatment of post platinum-based chemotherapy or for cisplatin-inelegibile patients. More precisely, monoclonal antibodies that target programmed cell death protein 1 (PD-1), (nivolumab and pembrolizumab), and monoclonal antibodies that target ligand of PD1 (PD-L1) (atezolizumab, durvalumab and avelumab) have been FDA approved for mUBC [36–40]. The PD-1/PD-L1 pathways have a critical role in the tumor escape from immune system (Fig. 1). The interaction between PD-1, expressed on the surface of activate T lymphocytes, and PD-L1, expressed on the surface of tumor cells, reduces the effector functions of T cells, preventing the attack of cancer cells by the immune system. Thus, by inhibiting the PD-1/PD-L1 interaction and transduction pathway, the monoclonal antibodies targeting PD-1 or PD-L1 are able to promote T-cell activation enhancing an immune response against the cancer cells [41]. Unfortunately the immunotherapy is not always effective and combinatorial strategy can be approached to enhance its efficacy [42, 43]. Recently, several studies have reported that defects in DDR genes could be potential predictive biomarkers of clinical response to immune checkpoint inhibitors in several type of tumors including metastatic urothelial bladder cancer [44–46]. The DDR pathway preserves genomic stability and defects in one of components of DDR network can lead the accumulation of DNA damage increasing the tumor mutational burden. The acquired somatic mutations result in the generation of neoantigens, presented on the cancer cell surface through the major histocompatibility complex (MHC) class I molecules and able to trigger the activation of cytotoxic T-cells [47–49]. Therefore, the high tumor mutational burden makes the cancer cells more immunogenic and thus able to elicit an antitumor immune response. Interestingly, the accumulation of DNA damage, that arises from the loss of the ability to repair the DNA repair, can results in the activation of the stimulator of interferon genes (STING) pathway, an innate immune signalling activated by cytosolic DNA usually during a viral infection, that has an important implication also in tumor detection (Fig. 1) [50]. The STING pathway leads to type I interferons (IFNs) production which, by acting in autocrine or paracrine manner, results in the activation of an anti tumor immune response. Moreover, the activation of STING pathway also leads to increased expression of PD-L1 on the cancer cells [51–53].

Interestingly, the DDR inhibitors, as PARP inhibitors, determine an increase of DNA damage, especially in tumors that show a defects in DNA repair pathways, increasing the tumor mutational load and stimulating the immune recognition of the cancer cells [54]. At the same time, in addition to induce DNA damage, PARP inhibitors cause the adaptive upregulation of PD-L1 expression with immunosuppressive effect [55]. These biological evidences motivate the combination of PARP inhibitors with immune checkpoint inhibitors, particularly the use of antibodies that target the receptor PD-1 and its ligand PD-L1, for the treatment of different type of tumors, as shown by the various launched clinical trials (NCT03167619) (NCT02861573) (NCT03338790) (NCT03330405). Therefore, the PARPi/ICIs combination may represent an attractive therapeutic strategy also for the treatment of patients with metastatic urothelial bladder cancer. Indeed, an open-label randomized multi-drug biomarker-directed phase Ib study, the BISCAY trial, is under way to evaluate the effects of the treatments with the PARPi Olaparib as a single agent therapy, or in association with the immune checkpoint inhibitor durvalumab (anti PD-L1), for treatment of mUBC patients who have progressed on prior treatment and also presented defects in DNA-repair genes (NCT02546661) [Table 1]. Besides anti-PD/PD-L1 therapy, ongoing clinical trials are also investigating the safety of adoptive T cell therapy in bladder cancer. Two phase I trials are investigating the targeting of tumor associated antigen (TAA) in order to personalize the genetic engineering of patients immune cells to target the specific antigen. Moreover, the introduction of immune checkpoint inhibitors that target the cytotoxic T lymphocytes-associated protein 4 (CTLA4) has resulted in a real improvement [7]. Recently, the CTLA4 combination with DDR targeting has been introduced in some tumors [7, 56].

## Epigenetic drugs and PARP inhibitor could improve the immune-checkpoint efficacy in UBC treatment

### PARP inhibitors and epigenetic drugs combination

Epigenetics is defined as a heritable modifications to DNA without alteration in the nucleotide sequence, resulting in altered gene transcription and chromatin structure. Epigenetic changes include DNA methylation and post-translational histone modifications involving methylation or acetylation. Epigenetic marks require the activity of specific cellular enzymes to be generated and maintained: DNA methyl transferase (DNMT) for DNA methylation and the opposite activities of histone acetyl transferase (HAT)/histone deacetylase (HDAC) and histone methyl transferase (HMT)/histone demethylase for determining the status of histone acetylation and methylation, respectively. The cooperation between these enzymes ends up in the chromatin condensation that leads to gene silencing [57, 58] Epigenetic modifications are common in bladder tumors and involve genes responsible of chromatin modification and remodelling. Mutations in histone deacetylase genes, HDAC, ARID1a, SW1/SNF family genes and others have been reported at very high frequency in advanced bladder tumors [24, 59]. Therefore, the pharmacological targeting of these modifiers could be effective in bladder tumors carrying these mutations. HDAC inhibitors single agent, such as romidepsin, TSA and SAHA, have been recently reported to affect cell growth and proliferation of 5637 bladder cancer cell line. These drugs determined cell death by modulating the expression of proteins involved in cell cycle progression, apoptosis, autophagy, reactive oxygen species generation and DNA damage repair [60]. However, while preclinical studies have shown encouraging results by using epigenetic drugs, uncertain outcomes have derived from the introduction of epigenetic drugs in clinical trials. Interestingly, TCGA analysis has documented that 75–90% of bladder tumor have a modification that affect epigenetic modifiers. Pharmacological targeting of these modifiers could be beneficial in patients whose tumors have these mutations. A concomitant defect in HR-DNA repair gene may indicate personalized treatment by combining the epigenetic drugs with PARP inhibitors [24, 25].Recently, the DNA methyltransferase inhibitors (DNMTi) and PARP inhibitors (PARPi) have been reported to act synergistically to induce cell death in in vitro and in vivo models of acute myeloid leukemia (AML), breast and ovarian cancers [61–63]. The most widely used DNMTi are the cytosine analogues 5-azacytidine (Aza) and 5-aza-2′-deoxycytidine (Decitabine) [64]. The cytosine analogues are incorporated into DNA during replication leading to formation of DNMT-DNA adducts that inhibit the catalytic activity of DNMT1 and trigger its degradation leading to global DNA demethylation [65, 66]. Besides epigenetic effects, the DNMTis are also able to increase the PARP-1 trapping at the DNA damage sites enhancing the DSBs cytotoxic effects induced by PARP inhibitors (Fig. 1) [62]. In fact, PARPi by inhibiting the catalytic domain of PARP-1 prevent its PARylation and then its release from the DNA damage site generating PARP-DNA complexes that result in the formation of cytotoxic DSBs [67, 68]. Moreover, the DNMTis, in addition to increase the PARP1 retention at the DNA damage site, also enhances the intracellular levels of reactive oxygen species (ROS) [63]. The accumulation of ROS induces PARP activation, in a cAMP/PKA-dependent manner, which ends in an amplified sensitivity of cancer cells to PARP inhibitors [69, 70]. Based on these findings, the use of DNMTi have been suggested to enhance the efficacy of PARPi in producing DSBs cytotoxicity.

### Epigenetic drugs and immune checkpoint inhibitors combination

The epigenetic alterations are used by cancer cells also to escape from the host immune system [71]. The immunoevasion is among the major obstacles to further improve the efficacy of cancer immunotherapies and to increase long-lasting disease control. Several epigenetic drugs able to revert the epimutations are available and some of them are also approved for clinical use.

The epigenetic drugs exert an immunomodulatory activity that leads to more effective recognition of cancer cells by immune system [72]. Indeed, the epigenetic drugs can enhance the immune response against the cancer cells through different mechanisms including the activation of viral defense pathway. In particular, demethylating agents can activate a viral defense pathway as result of stimulation of expression of endogenous retroviral sequences (ERVs). This mechanism, known as “viral mimicry”, drives the immunogenicity of cancer cells and enhance the immune signaling [73, 74]. The immunomodulatory action of epigenetics drugs provides a strong rationale for their clinical use in combination with immune checkpoint inhibitors [75, 76].

Interestingly, RRx-001, that is a new DNA damage inducer, but also an epigenetic and immunomodulatory drug, has been recently investigated as single chemotherapeutic agent able to to re-sensitize tumor to prior therapy [77–79]. RRx-001 has also been reported to prime tumors to respond to immunotherapy and it has been included in several clinical trials of phase II (NCT02096354, NCT02489903, NCT024529). The low toxicity profile of RRx-001 differentiates this agent from standard anticancer drugs, such as chemotherapeutics, targeted small molecules inhibitors, radiation, epigenetic agents and checkpoint inhibitors [79, 80].

The anticancer agent RRx-001 acts by inducing the accumulation of reactive oxygen and nitrogen species in the hypoxic tumor microenvironment activating the DNA damage response via phosphorylation of histone H2AX (γH2AX), induction of ATM and p53 [80]. Moreover, RRx-001 is able to induce the reduction of DNMTs activity, probably as a result of the oxidation of important residues of cysteine present on enzymes. The reduction of DNA methylation levels, induced by RRx-001, triggers the viral mimicry mechanism by the transcription of epigenetic silencing endogenous retrovirus (ERVs) inducing an antitumor immune response [78, 79]. Indeed, RRx-001, in urothelial bladder cancer cells, is able to trigger DNA damage response, to reduce the DNMT1 levels and to increase the transcriptional levels of the interferon type III and the interferon stimulated genes (ISGs) (Morra F, Merolla F, Criscuolo D et al., JECCR, accepted for publication). The ability of RRx-001 to trigger the DNA damage response, and also to act as immunomodulatory agent, is congruent with the use of the epigenetic agent RRx-001, which enhances the sensitivity to immune checkpoint inhibitors, with PARP-inhibitors in bladder cancer.

## Conclusion

Recently, the possibility to combine epigenetic agents and immune checkpoint inhibitors to optimize the PARP inhibition has been explored, mostly to overcome the PARP-inhibitors resistance [81, 82]. By limiting the overexpression of PD-L1 with epigenetic agent, the combination of PD-L1 inhibitors and PARPi might lead to the immunogenic cell death of cancer cells, offering a therapeutic strategy based on the synergic effect of drugs which prime tumors and overcome resistance. A combined treatment can also be conceived to prevent cancer stem cell resistance to PARPi by adding the DNMT1 and HDAC inhibitors as performed in few preliminary studies [83].

Drugs targeting the DDR process have been studied in combination with compounds that act epigenetically to modulate the expression of genes involved in the DDR in cancer [61, 83], enhancing their effects. Indeed, the histone lysine methyl transferase (HKMT) inhibitors may prevent the retention of BRCA1/BARD1 complex at DSBs sites promoting the NHEJ repair and enhancing the effects of PARP inhibitors [82, 84]. Finally, in mUBC preclinical model it has been recently reported a strong reduction of doses, with the maintenance of efficacy, by combining the DNA damage inducer RRx-001 with PARP-inhibitor olaparib that is expected to determine a strong improvement of the efficacy of the immune checkpoint inhibitors.

In conclusions, the PARP inhibitors drugs as single mode therapy or in combination with standard therapy are in clinical trials for mUBC with a DDR deficient background. This approach is likely to represent a new rationale for combined therapeutic strategies. However, besides specific gene traits, additional biomarkers to establish appropriate drugs usage are missing. Preclinical investigations suggested that UBC cells with low levels of CCDC6 perform as BRCA-like defective cells with sensitivity to small molecule inhibitors of the repair enzymes PARP1/2. Moreover, in high grade UBC the identification of two clusters of patients based on CCDC6 and USP7 expession can possibly suggest the combination of DDR targeting with DNA damage inducer RRx-001 which may highly improve the efficacy of immune checkpoint inhibitors reducing the doses and the side effects [85].

## Abbreviations

ARID: AT-rich interaction domain containing
ATM: Ataxia telengiectasia mutated
ATR: Ataxia telengectasia and Rad3 related
BARD: BRCA1 Associated RING Domain 1
BC: Bladder cancer
BER: Base excision repair
BRCA: Breast cancer
BRIP1: BRCA1 Interacting Protein C-terminal Helicase 1
cAMP: Cyclic Adenosine Monophosphate
CCDC6: Coiled coil domain containing 6
CHK1: Checkpoint kinase 1
CHK2: Checkpoint kinase 2
DDR: DNA damage response and repair
DNMT: DNA Methyl transferase
DSBs: Double strand breaks
ERVs: Endogenous retroviral sequences
FANC: Fanconi anemia
FANCF: FA Complementation Group F
FDA: Food and drug administration
HAT: Histone acetyl transferase
HDAC: Histone deacetylase
HKMT: Histone lysine methyl transferase
HMT: Histone demethylase
HR: Homologous recombination
HRD: Homologous recombination defects
ICIs: Immune checkpoint inhibitors
LOH: Loss of heterozygosity
MDC1: Mediator of DNA Damage Checkpoint 1
MHC: Major histocompatibility complex
MIBC: Muscle invasive bladder cancer
mUBC: Metastatic urothelial bladder cancer
NAD: Nicotinamide adenine dinucleotide
NHEJ: Non-homologous end joining
NMIBC: Non muscle invasive bladder cancer
PALB2: Partner and localizer of BRCA2
PARP: Poly (ADP-ribose) polymerase
PD-1: Programmed cell death protein 1
PD-L1: Ligand of PD1
PKA: Protein Kinase A
PP4c: Catalytic subunit of serine-threonine protein phosphatase 4
ROS: Reactive oxygen species
SSBs: Single strand breaks
STING: Stimulator of interferon genes
SW1/SNF: SWItch/Sucrose Non-Fermentable
UBC: Urothelial bladder cancer
